# Consequences of bariatric surgery on outcomes in rheumatic diseases

**DOI:** 10.1186/s13075-019-1869-z

**Published:** 2019-03-28

**Authors:** Eric Lespessailles, Emneh Hammoud, Hechmi Toumi, Nada Ibrahim-Nasser

**Affiliations:** 1 EA 4708 I3MTO Laboratory, University Orleans, 45067 Orleans, France; 2 Department of Rheumatology, Regional Hospital of Orleans, 14 avenue de l’hopital, CS 86709, 45067 Orléans Cedex 2, France; 3 Department of Physical Education, University of Balamand, EL-Koura, Lebanon

**Keywords:** Obesity, Bariatric surgery, Rheumatoid arthritis, Osteoarthritis, Gout, Psoriatic arthritis, Fibromyalgia, Low back pain, Osteoporosis

## Abstract

Obesity is associated with numerous comorbidities including some rheumatic conditions. Through adipose-derived inflammation, obesity has been shown to induce increased initiation, progression, and worse responses on outcomes of rheumatic diseases. Bariatric surgery is being increasingly used thanks to its positive effects on major comorbidities such as type 2 diabetes mellitus and hypertension. Consequently, surgically induced weight and adipose tissue losses might play a role in the course of rheumatic conditions. The present narrative literature review aims to provide rheumatologists with an update on both the positive and negative effects of bariatric surgery on the rheumatic outcomes reported in the literature. Current evidence seems to show improved outcomes in obese populations with rheumatic disorders after bariatric surgery. However, rigorous prospective controlled studies with long follow-up are needed. Bariatric procedures have deleterious effects on bone and are associated with an increased risk of fractures.

## Background

The epidemic of obesity is a well-established phenomenon worldwide. According to the World Health Organization, as much as 35% of the population worldwide is obese or overweight. In Europe, among middle-aged people, its estimated prevalence is about 15 to 20%.

For a long time, adipose tissue was considered as an amorphous tissue. Yet, there is accumulating evidence that white adipose tissue is a highly dynamic organ, a real endocrine organ, which is now considered as both one of the major actors of normal metabolic function and an orchestrator of energy homeostasis through bone and fat cross-talk.

Obesity, a complex chronic disease of the fat tissue, is often associated with other diseases, including rheumatic diseases. Therefore, a better knowledge of its interactions with the musculoskeletal system is of high clinical relevance [1]. The dramatic weight loss observed with bariatric surgery might impact musculoskeletal diseases.

In view of the rocketing rise in bariatric surgery (BS) procedures, in this article, we summarize the current clinical investigations reporting the relationship between BS-induced weight loss and rheumatic diseases.

Indeed, contrasting with the benefits associated with significant and durable weight loss on inflammatory rheumatic disorders, rheumatologists should consider the consequences of obesity but also the dramatic weight loss associated with malabsorptive procedures of bariatric surgery particularly as regards the risk of fragility fractures.

## Methods

Relevant studies discussed in this narrative review were identified via searches on PubMed and online journals and from the authors’ own libraries. Only peer-reviewed, English-language journals were included. The following search terms were used in various combinations: bariatric surgery, osteoporosis, osteoarthritis, rheumatoid arthritis, psoriatic arthritis, gout, adipokines, fracture, bone densitometry, obesity, and low back pain. There was no restriction on the date of publication. The original articles and abstracts resulting from the search of database were screened by three reviewers independently (E.L, E. H, N.I.N). Case reports were not selected. Whenever possible, priority was given to evidence from randomized control trials or meta-analyses. References from the articles retrieved and publications in the authors’ library were also used.

### Bariatric surgery: indications and procedures

The sole long-term effective weight loss option for people with obesity and severe associated comorbidities is bariatric surgery. As a result of the development of the laparoscopic approach, since 2005, the number of open operations has been outnumbered by laparoscopic operations [2]. Another significant evolution in the field of BS is the increasing use of laparoscopic sleeve gastrectomy and the declining use of laparoscopic adjustable gastric banding. Although BS indications depend on the specific recommendations of international learned societies, usually BS can be envisaged in patients with a BMI > 40 kg/m^2^ or with a BMI ≥ 35 kg/m^2^ and associated comorbidities, such as hypertension, type II diabetes mellitus, or obstructive sleep apnea, which may improve with sustained excess weight loss. In the present review, we describe three procedures mainly used with the coelioscopic approach.

Laparoscopic adjustable gastric banding (LAGB) is performed by placing an adjustable silicone band ring under the cardia, thus creating a small gastric pouch (around 15 ml). The ring is connected with a port which permits its adjustment by a percutaneous punction. This is a purely restrictive procedure; the associated excess weight loss observed in patients with a long follow-up is about 40 to 60%.

Laparoscopic sleeve gastrectomy consists in the resection of 75–80% of the stomach removing the fundus and a significant part of the gastric body. Its mechanism of action is restrictive in that it leaves a small tubular stomach (100–150 ml); however, it should be noted that as a result of the resection of the gastric fundus, ghrelin production is dramatically decreased, thus also inducing an appetite-regulating hormone action [3]. With this surgical technique, the excess weight loss observed is around 60 to 70%.

Laparoscopic Roux-en-Y gastric bypass (RYGB) also involves resection of the upper part of the stomach from the rest, creating a new stomach pouch (around 20 ml). The distal end of the jejunum is connected to the new gastric pouch by a gastro-jejunostomy. This ROUX-limb is usually 1.50 m long; the longer this alimentary limb is, the higher the malabsorption. The biliopancreatic limb is connected to the Roux-limb. This technique combines both malabsorptive and restrictive mechanisms of action with the small gastric pouch. It leads to an early effect on satiety but also metabolic effects thanks to changes in neuro-endocrine gastro-intestinal hormones induced by the digestive tract rearrangement.

### Bariatric surgery and osteoporosis

There is substantial evidence that BS procedures are associated with a negative effect on bone health [4]. Several prospective studies have shown that BS is associated not only with an increase in bone turnover markers, in favor of an increased resorption, but also with a decrease in bone mineral density (BMD). It is clinically relevant to distinguish among the BS procedures those which are purely restrictive and those which combine restrictive and malabsorptive techniques, since a greater increase in bone turnover markers and in BMD loss is associated with malabsorptive procedures [5]. In turn, these accelerated and profound BMD losses increase skeletal fragility.

In the seminal study conducted in the UK, no significant increase in fracture risk associated with bariatric surgery procedures was shown, probably linked to the short duration of follow-up [6].

There is an increased risk of overall fracture in patients who have undergone bariatric surgical procedures [7]. However, further research is required to determine which procedures are really associated with this condition, at which time course, in what period it occurs, and which specific bone sites are affected. The literature provides some clues on these issues as in all the studies where a separate analysis of restrictive and malabsorptive procedures was conducted [8], malabsorptive procedures were shown to be associated with an increased risk of fracture [8–10], whereas LAGB was not. One study reported a trend of increased fracture risk as early as 1 to 2 years after bariatric surgery [9]. Results concerning the use of sleeve gastrectomy in this setting are inconclusive due to its more recent use and the lack of prospective studies with sufficient follow-up. To the best of our knowledge, only two studies have shown that the risk of fracture after BS might be bone site specific with a higher susceptibility for upper limb fracture [8].

### Bariatric surgery and osteoarthritis

Obesity is one of the crucial risk factors for the initiation and progression of osteoarthritis (OA), particularly at the knee joint. Body mass index is theoretically a modifiable risk factor associated with OA. However, although the inflammation and metabolic effect of obesity on hand and knee OA has been well recognized, evidence for the relationship between obesity and hip OA remains weaker and inconsistent.

Although, in the cross-sectional analysis of the Rotterdam study, no intermediate effect of metabolic factors was added to the effect of overweight on hand osteoarthritis [11], recent data from the Netherlands Epidemiology of Obesity study, investigating the respective roles of mechanical and systemic stress on hand OA in a large cohort, showed a significant association between hand OA and metabolic syndrome after OR adjustment: 1.46 (95% CI, 1.06–2.02) [12]. In a systematic review of the literature on bariatric surgery, knee complaints were also recorded [13]. Results showed that BS was associated with an antalgic effect in individuals with symptomatic knee OA [13]. BS may also benefit obese patients with knee or hip osteoarthritis [14]. However, the paucity of data and the lack of randomized controlled trials were highlighted by the authors.

### Bariatric surgery and rheumatoid arthritis (RA)

Underlying the relationship between obesity and RA, there are some unsolved questions and paradoxes [15, 16]. Obesity might be considered as one of the crucial risk factors for the initiation and progression of rheumatoid arthritis [17, 18]. This point, however, is still debated, particularly the apparent association between seronegative RA and obesity and whether age and gender might play a role in this relationship. In addition, being obese in early RA decreases the chance of achieving good disease control: a meta-analysis showed that the odds of achieving remission were reduced by 43% and the odds of achieving sustained remission were reduced by 51% [19]. Furthermore, in RA treatment, the response rate to anti-tumor necrosis factor was reduced in obese patients and more generally obesity was associated with worse RA disease outcomes.

In this setting, Sparks et al. [20] conducted an investigation into RA outcomes in RA patients subjected to bariatric surgery. Fifty-three RA patients were assessed on RA characteristics and disease activity following a bariatric surgery procedure (mostly RYGB, 81%) with a mean ± SD time of most recent follow-up visit of 5.8 ± 3.2 years after surgery [20]. In this cohort, at 12 months following surgery, 6% instead of 57% at baseline had moderate to high disease activity (*p* < 0.001). Furthermore, 74% of RA patients compared to 26% at baseline were in remission at the most recent follow-up visit. Concerning the observed improvement in RA outcomes, the respective roles of excess weight loss per se (70% mean excess weight loss in this study) and other metabolic effects are unclear because many factors that were not considered in this study may contribute to this improvement, including changes in adipokines [1] and in gut hormones [21] and alterations in the microbiome after bariatric procedures [22].

### Bariatric surgery and psoriatic arthritis (PsA)

PsA patients have a higher prevalence of obesity than patients with psoriasis only [23] with an OR of 1.58 (95% CI 1.19–2.09). In fact, obesity is not only a comorbid condition in PsA but a real risk factor. This has been demonstrated in two large prospective studies, one using the US Nurse Health study II including 89,049 women [24], the other one using a database of medical records representative of the general UK population [25]. Similar results were observed in the two different populations, i.e., among patients with psoriasis, a high BMC linearly increased the risk of developing PsA. In addition, obesity might play a role in the severity of the PsA and interfere with the capacity to achieve and sustain minimal disease activity [26].

Furthermore, it was demonstrated that a higher than 5% weight loss against the baseline body weight was associated at 6 months with a higher rate of achieving minimal disease activity (MDA), and a higher than 10% weight loss from baseline was associated with an OR of achieving MDA of 6.67 (95% CI 2.41–18.41), *p* < 0.001 [26]. Taken together, these results showed the potential interest of the sustained and profound weight loss induced by BS on PsA.

One retrospective monocentric study assessing the effects of surgical weight loss including 86 patients with psoriasis and 21 PsA has been conducted [27]. The percentage of patients with a reduction of > 5 on a rating scale 0–10 (between before surgery and 1 year post surgery) was the primary end point of the study. In the 21 patients who had PsA, disease severity rating decreased from 6.4 to 4.5 (*p* = 0.01); a more clinically relevant decrease was observed in patients with the worst disease (8.2 vs 4.8) (*p* < 0.01). The sample size in this study did not permit any definitive conclusions although results are encouraging.

In the population-based health registry of Denmark, data were investigated in order to evaluate the first occurrence of PsA in patients treated with gastric bypass or gastric banding [28]. A decreased risk of PsA (adjusted HR, 0.29; 95% CI 0.12–0.71) was found in patients who underwent a gastric bypass, but this was not observed in patients following gastric banding (adjusted HR, 0.53; 95% CI 0.08–3.56) [28].

### Bariatric surgery and gout/hyperuricemia

Many epidemiologic studies have underlined the association between obesity and hyperuricemia. A dose-dependent effect has been demonstrated between the increase in BMI and the risk of incident gout.

Many learned rheumatology societies recommend weight loss for gout management in obese subjects, as weight loss has been found to both decrease uricemia levels and reduce the incidence of gouty arthritis. Thus, it is not surprising in this setting that bariatric surgery, which is increasingly called “metabolic surgery”, has been evaluated to demonstrate improvement in metabolic parameters such as hyperuricemia.

Although one of the seminal studies, conducted on Swedish obese subjects, concluded that BS prevented gout and hyperuricemia [29], this study was not a randomized controlled trial, the participants included were mainly women (70%) and gout incidence was not a predefined endpoint [48].

However, interestingly, in a study by Sjöström et al. [30], the higher the weight loss observed, the lower the serum uric acid level obtained. Long-term studies on gout incidence in patients with a previous history of gout incidence who underwent BS are very scarce.

Recently, a systematic review of longitudinal studies investigated the effect of weight loss in overweight or obese individuals with gout [31]. A weight loss of > 3.5 kg showed beneficial effects on gout attacks at medium/long-term follow-up based on six studies including only two trials with bariatric procedures [31]. Rigorous, preferably randomized controlled, trials are mandatory in this field of investigation due to the low methodological quality of the eligible studies included in this systematic review.

### Bariatric surgery and systemic lupus erythematosus (SLE)

Between 2005 and 2013, in a retrospective monocentric study, 31 patients with SLE were investigated to study the bariatric surgery outcomes related to their rheumatic disease [32]. In the 3 years post-surgery (mostly RYGB), 42% of patients were able to decrease their number of immunosuppressive drugs and even to stop glucocorticoid in 19.3% of cases. However, major postoperative complications associated with the perioperative use of immunosuppressive drugs were encountered and the early (30 days) complication rate was 13%.

### Bariatric surgery and fibromyalgia

There is also evidence that obesity is associated with musculoskeletal pain in other conditions than OA [33]. In a large cross-sectional study including more than 6000 middle-aged women, obesity was independently associated to musculoskeletal pain [34]. This significant association between joint pain and obesity and the potential improvement of pain following weight loss induced by exercise was investigated in 33 participants with widespread chronic pain, 79% of them fulfilling the fibromyalgia classification criteria. Among this small population, 21% were obese but there were no specific data reported in this work to assess the relationship between pain improvement and weight loss [35]. Behavioral weight loss has been also suggested to constitute a promising nonpharmacological treatment to manage fibromyalgia.

Consequently, in a retrospective study, patients with fibromyalgia who underwent laparoscopic RYGB were assessed in pre- and postoperative conditions about their pain score [36]. Although a significant decrease in median pain scores (from 9 to 3) was found, the limited number of patients (*n* = 10) and the retrospective design of the study did not permit any strong conclusions about the benefit of BS in the management of fibromyalgia in obese populations [36]. In addition, the assessment of pain in obese individuals may be a difficult task due to altered pain perception and processing [37].

### Bariatric surgery and low back pain

Among the obese population, low back pain (LBP) is a frequent complaint, due to abnormal mechanical loads placed on the spine and other biopsychosocial parameters.

The relationship between obesity and LBP is generally accepted, but the mechanisms of this association are not well elucidated. Studies have shown no association between low back-related problems and BMI [38, 39].

Nevertheless, several authors have investigated the effects of bariatric surgery on outcomes related to musculoskeletal conditions in obese populations including the effects on physical function and activity [40], joint pain [41], and health-related quality of life [41]. More specifically in patients with chronic LBP, the effects of BS, namely vertical banded gastroplasty, were evaluated on their functional status before and 2 years after surgery [42]. In the 29 morbidly obese participants, there was an improvement in their functional disability as reflected by significant decreases in the scores of the Rolland-Morris Disability questionnaire, the Oswestry LBP Disability questionnaire (ODI), and the Waddell Disability index. The weight reduction obtained following this surgery was also associated with a significant decrease in LBP assessed by the visual analogue scale (VAS) [42].

Another study including 38 patients who completed both pre- and postoperative ODI and VAS at 12 months showed that patients demonstrated a mean 44% decrease in LBP on the VAS (*p* = 0.006) [43]. In addition, a large majority of patients (68.4%) improved their VAS scores while 13% and 18.4% respectively remained stable or deteriorated. No significant associations were found between weight loss and VAS or ODI score [43]. In contrast to these two uncontrolled studies, a prospective controlled study confirmed the interest of performing BS in morbidly obese patients to induce favorable changes in pain and quality of life [33]. In this study, within 3 months, there was a significant decrease in low back and knee pain in the BS group (*n* = 25) but not in the control group (*n* = 20) (*p* = 0.021) [33]. Furthermore, after surgery, the percentage of patients having moderate to severe LBP decreased from 75 to 38.9%, demonstrating rapid improvements in LBP as early as 3 months post-surgery. Contrasting with this improvement in joint pain symptoms, at 3 months, 44.4% and 33.3% of patients in the surgery group could not respectively climb one flight of stairs or walk several blocks [33]. Another uncontrolled study showed an antalgic effect of weight loss on both axial back pain and radicular leg pain 1 year postoperation in 30 morbidly obese adults [44]. Although no significant correlation was found between the delta of weight reduction and the delta in back pain improvement or disc space height, disc height changes were significantly different at baseline versus 1 year postoperation (6.8 ± 1.3 mm to 8.8 ± 1.5 mm; *p* < 0.001) [44].

### Rheumatologic side effects of BS

Acute attacks of gout have been observed after BS [45]. In the immediate postoperative period, a more rapid and larger decrease in the serum uric acid level may trigger gout attacks [46].

In addition to gouty attacks and the negative effects on bone health reported above, BS procedures were associated in the 1980s with postoperative arthropathy. Arthritic syndromes were associated with jejuno-ileal bypass surgery, increased antigenic stimuli favored by changes in the microbiota from the small bowel blind loop [47]. A permanent remission of arthropathic symptoms was obtained after dismantling of the bypass [48]. The inflammatory arthropathy-like syndrome was described as a non-erosive seronegative polyarthritis which developed after jejuno-ileal bypass. Circulating immune complexes [49] and the dissemination of byproducts from the excluded bowel [47] were incriminated as physiopathogenic processes. One case report described the occurrence of a spondyloarthritis-like syndrome after partial gastroplasty; a complete resolution of the symptoms was obtained after the use of sulfasalazine and methotrexate [50].

Finally, bypass surgery has also been associated with rare joint and skin manifestations due to immune mechanisms resulting from small bowel bacterial over growth occurring in cases of blind intestinal loop, known as the bowel-associated dermatitis-arthritis syndrome [51].

## Conclusion

Main details of relevant studies clarifying the effects of bariatric surgery in patients with rheumatic diseases are reported in Table 1**.**

**Table 1:** Basic characteristic and rheumatic disease outcomes after bariatric surgery in selected studies with their main limitations

Authors	Patients/bariatric surgery/follow-up	Rheumatic diseases	Outcomes	Main limitations
Lalmohamed et al. [6]	*n* = 2079 pts *n* = 10,442 controls 60% LAGB, 29% RYGB 2.2 years	Osteoporosis	No increase in OP fracture rate	Retrospective design Limited follow-up duration
Zhang et al. [7] *(meta-analysis)*	Five observational trials and one RCT but in pts with T2DM ≠ BS 2.2 to 4.8 years	Osteoporosis	Higher risk for any type of fracture in the surgical group Fracture risk in non-vertebral sites: 1.42; 1.68 in the upper limbs	Mainly retrospective and observational studies Comparison between only mixed and restrictive procedures
Rousseau et al. [8]	*n* = 9300 pts *n* = 38,028 sex-matched obese pts *n* = 126,760 non-obese controls 41% LAGB, 27% SG, 9% RYGB, 21% BD 4.4 years	Osteoporosis	Postoperative adjusted fracture risk higher in the bariatric group aRR: 1.38 A site-specific effect is suggested	Retrospective nested case control study Bariatric and obese groups not matched for BMI
Lu et al. [9] *(nationwide cohort study)*	*n* = 2064 pts *n* = 5027 propensity score-matched subjects 14% malabsorptive procedures, 86% restrictive procedures 4.8 years	Osteoporosis	Increased risk of fracture adjusted HR 1.21; malabsorptive procedures aHR 1.48	Retrospective design Data on BMI pre-surgery and post-surgery were not available.
Nakamura et al. [10]	*n* = 258 pts 94% RYGB 7.7 years	Osteoporosis	A twofold increased risk of OP fracture	Retrospective uncontrolled design on review of medical records Non-standardized data
Groen et al. [13] *(systematic review)*	13 studies *n* = 2286 pts *n* = 1551 non-surgical pts RYGB and LAGB 3 months to 6 years	Knee complaints in knee osteoarthritis	Overall significant improvement in knee pain was seen in 73% out of the used assessments	Mainly uncontrolled prospective studies No RCTs Limited follow-up duration
Gill et al. [14] *(systematic review)*	6 studies *n* = 2008 pts *n* = 1531 controls RYGB and LAGB 0.5 to 8 years	Knee and hip osteoarthritis	BS may benefit obese pts with hip or knee OA. Evidence that standardization of outcome is lacking	Inability to perform a pooled analysis or a meta-analysis due to lack of randomized controlled study
Sparks et al. [20]	*n* = 53 pts 81% RYGB 12 months and most recent follow-up	Rheumatoid arthritis	Decrease in disease activity and serum inflammatory markers Less RA-related medication use At 12 months, 6% of pts had moderate/high disease activity vs 57% at baseline	RUD SSS Validated measures not collected consistently
Sethi et al. [27]	*n* = 21 pts 91% LAGB 6.1 years	Psoriatic arthritis	Decrease in disease severity rating	Retrospective uncontrolled database analysis SSS Validated measurement not collected consistently Abstract
Nielsen et al. [31]	*n* = 132 pts ≠ BS 6 to 13 months	Gout	Low-quality evidence for gout attacks and achieving serum uric acid targets	A meta-analysis was not possible. The majorities of included studies were uncontrolled. The lack of rigorous studies is underlined.
Corcelles et al. [32]	*n* = 31pts 74% RYGB 3 years	Systemic lupus erythematosus	Decrease in SLE immunosuppression medication requirement	RUD SSS
Saber et al. [36]	*n* = 10 pts RYGB 24.5 months	Fibromyalgia	Decrease in median of pain score and points of tenderness	RUD SSS Phone interview
Khoueir et al. [43]	*n* = 58 pts 62% RYGB 12 months	Low back pain	44% decrease in axial back pain (VAS)	38 pts completed pre and post-op questionnaire SSS Limited follow-up duration Uncontrolled study
Lidar et al. [44]	*n* = 30 pts ≠ BS 12 months	Low back pain	Axial and radicular back pain decreased after surgery (VAS)	SSS Uncontrolled study
Vincent et al. [33]	*n* = 25 pts 72% RYGB 28% LAGB 3 months	Low back pain	54% reduction in mean score change in numeric pain rating scale	SSS Limited follow-up duration
Melissas et al. [42]	*n* = 29 pts VBG 24 years	Low back pain	Improved functional disability scores	SSS Uncontrolled study

*BD* biliopancreatic diversion, *BS* bariatric surgery, *LAGB* laparoscopic adjustable gastric banding, *OA* osteoarthritis, *OP* osteoporosis, *RA* rheumatoid arthritis, *RCT* randomized controlled trial, *RUD* retrospective uncontrolled design, *RYGB* laparoscopic ROUX-en-Y gastric bypass, *SG* sleeve gastrectomy, *SLE* systemic lupus erythematosus, *SSS* small sample size, *T2DM* type 2 diabetes mellitus, *VBG* vertical banded gastroplasty

While the excess weight loss induced by BS seems to produce significant and clinically relevant effects in decreasing pain in patients with knee osteoarthritis or chronic lombalgia, these findings require further confirmation in prospective controlled trials. BS also provides significant effects in lowering the level of uricemia although the evidence regarding the benefit of BS in obese individuals with gout is low. The relationship between the pathophysiology of RA and adipose tissue has not been fully elucidated but, following BS, RA outcomes appear to improve. Bariatric procedures have deleterious effects on bone metabolism, and there is a great body of evidence showing that this can lead to an increase in fracture risk, particularly in patients operated with malabsorptive techniques.

The present narrative literature review aimed to provide some new insights into the complex relationship between obesity and various rheumatic diseases and underlines the need to conduct additional trials, if possible long-term prospective controlled trials, to fully assess the risk-benefit ratio of BS in morbidly obese patients with rheumatic diseases.

## Abbreviations

BMD: Bone mineral density
BS: Bariatric surgery
LAGB: Laparoscopic adjustable gastric banding
LBP: Low back pain
MDA: Minimal disease activity
OA: Osteoarthritis
ODI: Oswestry LBP Disability questionnaire
PsA: Psoriatic arthritis
RA: Rheumatoid arthritis
RYGB: Laparoscopic Roux-en-Y gastric bypass
SLE: Systemic lupus erythematosus
VAS: Visual analogue scale
